# Long-read sequencing for neurological disorders: opportunities, challenges, and future directions

**DOI:** 10.1007/s10072-026-09283-y

**Published:** 2026-08-01

**Authors:** Hanabi Geiger, Yutaka Furuta, B. Lakshitha A. Perera, Russell Stewart, Rory J. Tinker, John A. Phillips

**Affiliations:** 1 Department of Pediatrics, Division of Medical Genetics and Genomic Medicine, Vanderbilt University Medical Center, Nashville, TN USA; 2 Division of Genetic Medicine and Clinical Pharmacology, Department of Medicine, Vanderbilt University Medical Center, Nashville, TN USA; 3 Vanderbilt University School of Medicine, Nashville, TN USA; 4 Department of Medical Genetics and Genomics, Icahn School of Medicine at Mount Sinai, New York, NY USA

**Keywords:** Long-read sequencing (LRS), Neurogenetic disorders, Epigenetic and methylation profiling, Pacific Biosciences HiFi, Oxford Nanopore Technologies (ONT), Clinical genomics

## Abstract

**Background:**

Many neurological disorders (NDs) have a genetic basis, yet traditional diagnostic tools such as EEGs, EMGs, and neuroimaging primarily capture downstream manifestations. Although short-read sequencing (SRS) has advanced genetic diagnostics, significant gaps remain. Large repeat expansions, complex structural variants, mitochondrial variants, transcript splicing alterations, and epigenetic changes, all common contributors to NDs, are difficult to resolve with SRS.

**Methods:**

This review examines the capabilities of long-read sequencing (LRS) technologies in addressing these limitations. We evaluate studies leveraging LRS for genetic diagnosis in NDs and assess current barriers to clinical adoption, including technological, analytical, cost-related, and ethical considerations.

**Results:**

By producing read lengths of tens of kilobases or more, LRS enables detection of variant types often inaccessible to SRS. Recent work has demonstrated its power in conditions such as Duchenne muscular dystrophy, fragile X syndrome, spinocerebellar ataxias, and unresolved mitochondrial syndromes. These findings highlight the potential of LRS to substantially increase diagnostic yield in NDs. However, major challenges persist: the need for high-quality DNA, demanding analytic pipelines, limited access outside major research centers, high costs, and ethical concerns including equity and management of incidental findings.

**Conclusions:**

LRS offers advantages for identifying complex genomic contributors to NDs and holds promise for improving diagnostic accuracy. Nonetheless, key technical, logistical, and ethical barriers must be addressed before widespread implementation is feasible. This review outlines current strengths, limitations, and emerging applications of LRS to guide clinicians and researchers in understanding how the technology can be applied today and what is needed for broader adoption.

## Introduction

Neurological disorders (NDs) can affect the central nervous system (CNS: brain and spinal cord) and peripheral nervous system (PNS: nerves beyond the spinal cord, including the autonomic nervous system (ANS), which regulates unconscious functions) [1]. Classification of NDs includes neurodegenerative, neuromuscular, brain/spinal, and peripheral nerve disorders, but overlapping phenotypes, genetic heterogeneity and environmental factors often complicate diagnosis [2]. Current diagnostic tests, including EEG, electromyography, autonomic studies, MRI, and functional MRI: primarily assess symptoms but often do not identify the underlying genetic basis of a ND [1].

Because many NDs have a genetic basis, DNA sequencing has become increasingly important in their diagnosis. The American College of Medical Genetics (ACMG) recommends next-generation sequencing (NGS). NGS technologies have become the foundation of modern genomic diagnostics due to their high accuracy, scalability, and cost-effectiveness. In these approaches, genomic DNA is fragmented into short segments that are sequenced in parallel and computationally aligned to a reference genome. Short-read platforms support a spectrum of sequencing strategies, including targeted gene panels, whole-exome sequencing (WES), and whole-genome sequencing (WGS), each offering different tradeoffs in coverage, cost, and interpretability. Targeted approaches focus on predefined gene sets and are often sufficient when clinical presentation suggests a specific diagnosis, whereas broader approaches such as WES or WGS enable the identification of novel or unexpected variants in cases with unclear phenotypes [3].

However, technical limitations inherent to short-read sequencing (SRS) render this technique insufficiently sensitive to multiple key genetic changes relevant to NDs, such as short tandem repeats (STRs), structural variants (SVs), alternative splicing, and epigenetic changes [4]. These gaps are especially evident across major genetic mechanisms of NDs: repeat expansion disorders (e.g., spinocerebellar ataxias, oculopharyngeal muscular dystrophy, Neuronal Intranuclear Inclusion Disease, or C9orf72 ALS/FTD), SV-driven disorders (e.g., Duchenne muscular dystrophy, LAMA2-related muscular dystrophy, facioscapulohumeral dystrophy, and titinopathies), mitochondrial and leukodystrophy syndromes, splicing and isoform disorders (e.g., congenital central hypoventilation syndrome, developmental and epileptic encephalopathies), and conditions caused by methylation differences or haplotype context (including imprinting disorders and several neurodegenerative diseases). This SRS diagnostic gap highlights why new sequencing approaches without such blind spots are needed.

Long-read sequencing (LRS) addresses many of these limitations. Current platforms, namely Pacific Biosciences (PacBio) HiFi and Oxford Nanopore Technologies (ONT), can sequence DNA and RNA molecules tens of thousands to millions bases in length without fragmentation [5]. Here, we discuss the role of LRS in NDs to provide additional diagnostic information, improve the diagnostic rate, and reduce the amount of time required to make a molecular diagnosis. We highlight how LRS can enable accurate detection of SVs, STRs, allelic phasing, and mitochondrial genome analysis, capabilities that directly address long-standing limitations of SRS. Through case discussions of emerging clinical applications, we assess the diagnostic impact of LRS and its potential to transform genetic testing in complex NDs.

## Overview of long-read sequencing technologies

**Fig. 1:**
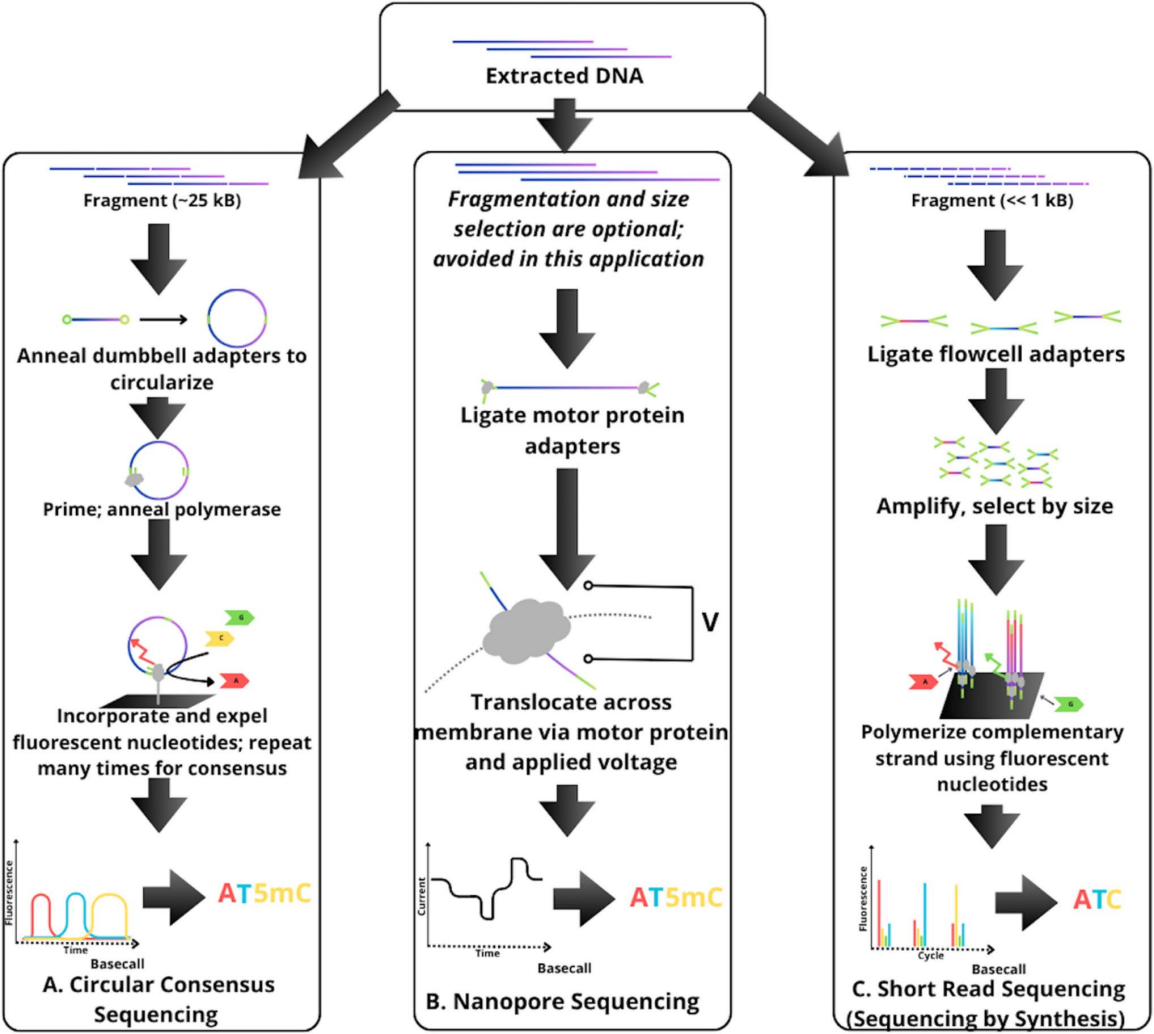
Comparison of high-throughput sequencing methods. **A**: In circular consensus sequencing (e.g. PacBio), the nucleic acids to be sequenced are fragmented into large fragments (dozens of kilobases in length), circularized, annealed with primers and polymerases, then sequenced. During sequencing, fluorescent nucleotides are repeatedly incorporated and expelled without elongating a daughter strand by means of an engineered polymerase. The circular sequence is read through many times to improve the signal-to-noise ratio. Base calling is then performed from fluorescence readings. Changes like methylation can be directly detected and read lengths are comparatively long, enabling better structural and epigenetic insight. **B**: In nanopore sequencing (Oxford Nanopore Technologies), sample nucleic acid fragmentation is not required. Motor protein adapters are ligated to sample nucleotides. Motor proteins translocate tagged nucleotides across semipermeable membranes. During base calling, traces of the transmembrane voltage over time are processed into corresponding nucleotide sequences. Again, direct methylation detection and long read lengths facilitate structural and epigenetic studies. **C**: In short read sequencing, also called sequencing by synthesis (Illumina), sample DNA is fragmented into comparatively short segments a few hundred base pairs in length. Adapters reverse-complimentary to adapters annealed to the flow cell service are then ligated to these fragments. Size selection and amplification are often performed before samples are introduced into the flow cell. In the flow cell, cluster amplification creates many identical clones of each sample sequence in close spatial proximity to improve the signal-to-noise of the fluorescent readout to come. Fluorescent oligonucleotides are incorporated, imaged, cleaved of fluorophores, and further extended in a cyclic process. The fluorescent signal observed during each cycle is then used for base calling. While epigenetic changes cannot be directly detected and read lengths are comparatively short, this method carries advantages of comparatively low cost and scalability

PacBio HiFi, released in 2019, uses *circular consensus sequencing (CCS)*. In this, DNA fragments are circularized and read through many times to create a consensus sequence output (Fig. 1 IIa). The resulting consensus achieves ~ 99.9% accuracy with read lengths up to ~ 25 kb [5]. With this method, HiFi avoids PCR amplification errors associated with SRS [6]. Additionally it enables accurate detection of STRs, SVs, and methylation changes, while also advancing ribosomal DNA and haplotype phasing analyses [5, 7].

ONT was launched in 2015 and can generate ultra-long reads (> 2 Mb) by threading single-stranded DNA or RNA molecules through nanopores, where base-specific current changes are measured in real time. There are three varieties of ONT sequencers currently available of which the smallest, the handheld MinION, has a read capacity of ~ 50 Gb (Fig. 1B) [8].

Both platforms require high–molecular weight DNA for optimal results, which can introduce practical limitations [9]. However, the platforms differ in other trade-offs (Table 1). HiFi has better per-read accuracy (~ 99.9%) but shorter read lengths (≤ 20–30 kb) and higher costs, limiting scalability. ONT can deliver much longer reads (> 2 Mb) and real-time sequencing at lower entry costs but it has higher raw error rates (~ 14%) [10]. Increasingly, hybrid approaches that combine HiFi’s accuracy with ONT’s read length are being used [7].

**Table 1:** Characteristics of PacBio HiFi and ONT

Feature	PacBio HiFi	ONT
Launch	HiFi/CCS chemistry: 2019. PacBio SMRT long-read sequencing platform commercially available since ~ 2011	~ 2015 (MinION public release)
Read length	Up to 20–30 kb	> 2 Mb (ultra-long reads; typical WGS reads shorter)
Accuracy	~ 99.9% (HiFi consensus after circular consensus sequencing). Raw single-pass accuracy lower (~ 85–87%)	~ 86% raw (R9 chemistry); R10.4 chemistry with consensus correction substantially improves accuracy. Direct comparison should use equivalent processing stages for both platforms
Key strengths	STR and SV detection, high consensus accuracy, allelic phasing, de novo assembly, native DNA sequencing (direct methylation detection)	Ultra-long reads, real-time sequencing, native RNA sequencing, broad base modification detection (DNA and RNA), cost-effective options, portable formats, polymerase-free chemistry performs well in GC-rich regions
Limitations	Higher platform cost, requires high-molecular-weight DNA (circularization step), limited base modification models compared to ONT, bias if input quality poor	Higher raw error rate (~ 14%), characteristic errors in homopolymeric sequences, read length may shorten with correction, high-molecular-weight DNA improves WGS performance (though not required for all applications), bias if input quality poor
Scalability	High-throughput instruments mostly in research centers; Vega offers lower-throughput benchtop option	Flexible: MinION (portable/field use), GridION (lower throughput benchtop), PromethION (high throughput). *Note: Flongle (low-cost single-use flow cell) was discontinued; R10 chemistry was not compatible*
Best applications	High-accuracy variant detection, phasing, de novo assembly, complex/dark genome regions, metagenomics, infectious disease	Ultra-long contigs, metagenomics, infectious disease, rapid or portable sequencing, epigenetics (both DNA and direct RNA)

## Advantages of LRS for neurological disorders (nds)

### Repeat expansion disorders

STRs can expand abnormally and cause NDs. Of the 47 currently known disease causing STR-associated genes, 37/47 (79%) primarily present with neurological symptoms [4].The most clinically relevant are *CAG* trinucleotide expansions (TNRs), which encode polyglutamine tracts that cause the resulting abnormal protein products to aggregate in the cerebellum and other brain regions. The CAG TNR alone has been reported to cause at least 10 NDs. Such TNRs underlie Huntington disease (OMIM#:143,100), spinal and bulbar muscular atrophy (OMIM#: 313,200), dentatorubral-pallidoluysian atrophy (OMIM#: 125,370), and several spinocerebellar ataxias. Expansions in other STR motifs cause fragile X syndrome (OMIM#:300,624), myotonic dystrophy (OMIM#:160,900), Friedreich ataxia (OMIM#:229,300), CANVAS (OMIM#:614,575), oculopharyngeal muscular dystrophy (OMIM#:164,300), and myoclonic epilepsy (OMIM#:601,068) [11].

SRS struggles to analyze tandem repeat regions due to polymerase stutter errors during SRS’ library amplification and sequencing, resulting in insufficient read length, misalignment, and miscalculation of repeat counts. Other confirmatory tests (Repeat-primed PCR, Southern blot) are thus frequently required to determine repeat count. In contrast, both PacBio HiFi and ONT can reliably resolve full repeat lengths, including GC-rich loci, to enable the detection previously missed or misclassified cases [11, 12].

The size limits of repeat detection differ substantially between SRS and LRS platforms, and these differences have direct diagnostic relevance. SRS reliably detects STR expansions only up to approximately 50–100 bp, corresponding to roughly 15–30 repeat units for trinucleotide repeats, beyond which read length, PCR stutter, and misalignment render accurate sizing unreliable. This means that pathogenic expansions in most repeat expansion disorders, which typically range from hundreds to thousands of repeat units, fall entirely outside the reliable detection range of SRS without supplementary methods such as repeat-primed PCR or Southern blotting. PacBio HiFi, with read lengths of up to 20–25 kb, can reliably span expansions of up to approximately 6,000–8,000 repeat units for trinucleotide motifs, covering the pathogenic range for the majority of known repeat expansion disorders including Huntington disease, most spinocerebellar ataxias, and Friedreich ataxia [4, 13, 14]. However, very large expansions such as those seen in myotonic dystrophy type 1, where CTG repeat counts can exceed 3,500 and the expanded allele may span tens of kilobases, and C9orf72-associated ALS/FTD, where GGGGCC hexanucleotide expansions can reach thousands of units, may approach or exceed HiFi’s read length ceiling, potentially preventing complete traversal of the largest alleles. ONT’s capacity for ultra-long reads exceeding 2 Mb means it is theoretically capable of spanning even the largest known pathogenic expansions in a single read, making it particularly advantageous for disorders with extreme repeat sizes. In practice, however, the higher raw error rate of ONT can reduce base-level accuracy within repetitive sequences, and achieving sufficient depth across expanded loci requires careful library preparation and sequencing design [15]. For most clinically relevant repeat expansion disorders, both platforms outperform SRS substantially, but platform selection should be guided by the anticipated expansion size, the repeat motif’s GC content, and whether precise interruption pattern characterization is required (Table 1).

While LRS demonstrates clear advantages in resolving full repeat lengths and interruption patterns across both GC-rich and complex loci, several practical constraints currently limit its routine clinical use for TNR disorders. First, LRS requires high-quality, high-molecular-weight DNA, which can be difficult to obtain from certain sample types such as dried blood spots, formalin-fixed paraffin-embedded tissue, or degraded archival specimens that are commonly encountered in clinical and research settings. This requirement is particularly relevant for repeat expansion disorders, where affected individuals may present late in disease course or where historical samples are the only available material [16]. Second, the per-sample cost of LRS remains substantially higher than established targeted approaches such as repeat-primed PCR or Southern blotting, which, despite their own limitations in repeat sizing accuracy, are widely available, reimbursable, and operationally familiar to clinical laboratories [13, 17]. Third, achieving reliable allele sizing for expanded repeats requires sufficient sequencing depth across the repeat locus, and the optimal read depth thresholds for different repeat motifs, locus architectures, and expansion sizes have not yet been systematically established across platforms. This is compounded by the fact that very large expansions, such as those seen in myotonic dystrophy type 1 or C9orf72-associated ALS/FTD, may approach or exceed the read length capabilities of some LRS platforms, potentially resulting in incomplete traversal of the expanded allele [15, 17]. Fourth, standardized, clinically validated high-throughput pipelines for repeat expansion calling using LRS are still in development, with most published workflows reflecting research-grade implementations rather than robustly validated diagnostic tools [18]. Together, these constraints mean that while LRS holds considerable promise for TNR diagnostics, its current deployment is best suited to cases where conventional methods have failed to yield a diagnosis, where repeat sizing beyond the range of standard assays is clinically necessary, or where simultaneous characterization of methylation status and repeat length is required.

### Structural variants (SVs)

**Fig. 2:**
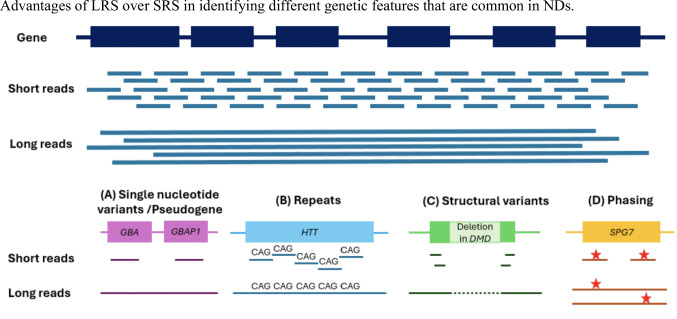
Sequencing Method Comparison of Variants. Advantages of LRS over SRS in identifying different genetic features that are common in NDs

SVs are genomic rearrangements ≥ 50 bp that disrupt regulatory architecture and gene expression. They include balanced inversions and translocations, unbalanced copy number variants (CNVs), and mobile insertions/deletions (Fig. 2) [19]. SVs have been implicated in Parkinson disease (OMIM#: 168,600), Alzheimer disease (OMIM#:104,300), ALS (OMIM#:105,500), autism (OMIM#:209,850), and ataxia syndromes [20–23]. Current analytic methods (karyotype, microarray, MLPA, SRS) often miss these variants [24]. However, LRS, with longer and more accurate single-molecule reads, enables better SV detection. ONT has revealed > 167,000 previously undetected SVs in population studies, and PacBio HiFi has identified clinically relevant SVs in disorders such as DMD (OMIM#:300,377), LAMA2 (OMIM#:607,855, 618,138), Kabuki syndrome (OMIM#:147,920), and Pelizaeus-Merzbacher (OMIM#:312,080) among others [25–31]. The detection of SVs using LRS improves up until 20 × coverage read depth. Assembling these SVs has similar computational demands to other LRS uses, although it is significantly less demanding than full genome assembly [32].

The resolution and detection capabilities of LRS for SVs differ meaningfully from SRS and also vary between LRS platforms. SRS-based SV detection is generally limited to variants larger than approximately 50 bp, the conventional minimum definition of an SV, but in practice SRS struggles to detect balanced SVs such as inversions and translocations, complex rearrangements, and variants in repetitive or segmentally duplicated regions regardless of size, due to short reads failing to span breakpoints. CNVs detected by SRS microarray analysis have a practical resolution floor of approximately 25–50 kb for array-based methods, though SRS WGS can detect smaller CNVs with sufficient depth. LRS substantially improves resolution across all SV classes [33]. PacBio HiFi can detect SVs as small as 50 bp with high precision due to its accuracy, and its read lengths of 20–25 kb enable reliable spanning of breakpoints in complex rearrangements and segmentally duplicated regions [34]. ONT’s ultra-long reads provide additional advantages for very large SVs and complex structural rearrangements such as chromothripsis, where multiple breakpoints must be resolved simultaneously across large genomic intervals. Certain SV classes benefit disproportionately from LRS. Mobile element insertions, which are largely invisible to SRS due to their highly repetitive sequences and the difficulty of mapping reads to insertion sites, are reliably detected by LRS. Inversions and balanced translocations, which produce no copy number change and are therefore missed by array-based methods and often misrepresented by SRS, are directly resolved by long reads spanning both breakpoints. Complex SVs involving nested or chained rearrangements, which are increasingly recognized as pathogenic in neurodevelopmental and neurodegenerative disorders, are similarly best characterized by LRS(Table 1) [35]. For neurological disorders specifically, the high prevalence of SVs in segmentally duplicated regions, including those relevant to conditions such as Charcot-Marie-Tooth disease, spinal muscular atrophy, and Pelizaeus-Merzbacher disease, makes LRS particularly impactful in this diagnostic context [25, 26, 31, 36].

### Phasing and allelic resolution

Conventional SRS merges maternal and paternal alleles into a consensus, obscuring cis/trans relationships between variants. Determining allele phasing is critical in autosomal recessive NDs, where pathogenicity depends on if the variants are on the same or opposite alleles. SRS can achieve phasing but often requires either sequencing both proband and parental DNA (trio sequencing) or using error-prone statistical assemblies (Fig. 2).

By spanning larger haplotype blocks, LRS enables direct phasing from a single individual’s DNA or RNA without requiring trio samples. PacBio HiFi in particular produces highly accurate phased haplotypes across complex regions that outperforms SRS and ONT [37, 38]. ONT relies on its length to detect many overlapping single nucleotide polymorphisms (SNPs) on heterozygote variants however it is outperformed due to PacBio’s accuracy [7]. This is specifically very useful for genes with paralogs (duplicated genes), which remain as one of the most challenging areas of molecular diagnostics due to limited differentiation ability (Fig. 2) [25].

### Mitochondrial genome sequencing

Mitochondrial DNA (mtDNA) is encoded by the circular mitochondrial chromosomes, which are multiploid (100–10,000 copies per cell) and include 37 essential genes. Variants in mtDNA are suspected to contribute to Parkinson disease (OMIM#:168,600), Alzheimer disease (OMIM#104,300), Huntington disease (OMIM#:143,100), and Kearns-Sayre syndrome (OMIM#:530,000) [39–42]. Pathogenesis of mtDNA-caused disorders often involves defects in the electron transport chain, oxidative stress, or large-scale deletions. SRS can detect single nucleotide variants (SNVs) but presents problems when trying to delineate mtDNA genes’ heteroplasmy, define structural rearrangements, or catch large deletions due to fragmentation and PCR artifacts [42]. While the protocol of mtDNA sequencing remains largely similar for SRS and LRS as it is with other DNA, LRS performs markedly better. This is because LRS can sequence an entire mitochondrial genome without having to align many smaller fragments, improving breakpoint resolution, heteroplasmy detection, and deletion discovery. In SRS these short fragments can be aligned incorrectly due to SVs, STRs, and the added challenges of heteroplasmy and nuclear mitochondrial DNA segments. For these reasons mtDNA is only sometimes reported with WGS, with targeted sequencing being the more relied on method for analyzing mitochondrial genomes [41]. LRS has successfully been used to detect Kearns-Sayre syndrome and Melas Syndrome [41, 43].

### Epigenetic disorders

Epigenetic modifications, especially DNA methylation, are important regulators of gene expression, and they are increasingly recognized as phenotypic modifiers in Mendelian disorders. Traditional methods such as bisulfite conversion of unmethylated cytosines into uracil, are limited by DNA degradation, bias, and the inability to resolve allele-specific effects in complex regions. The region of mtDNA being investigated varies the necessary depth, however the necessary depth is typically lower for LRS. LRS overcomes these issues by directly detecting base modifications during sequencing, PacBio HiFi through polymerase kinetics and ONT through changes in ionic current. The interpretation of these methylation signals can be computationally difficult with tools like Megalodon and Nanopolish being used This can determine single-molecule, allele-specific methylation status without DNA conversion or amplification [7, 44].

These capabilities are important in clinical applications. In fragile X syndrome (OMIM#: 300,624), LRS captures both *FMR1* repeat expansions and promoter hypermethylation, to detect mosaic methylation patterns linked to severity [45]. Imprinting disorders such as Prader–Willi syndrome (OMIM#:176,270) and Angelman syndrome (OMIM#:105,830) could benefit from simultaneous assessment of genetic variants and parent-of-origin methylation states by LRS [46]. The methylation status of the mitochondrial genes discussed also seems to have particular relevance, especially for Alzheimer disease [40]. Similarly, in Friedreich ataxia (OMIM#:229,300) and myotonic dystrophy (OMIM#160,900), LRS can resolve expanded alleles as well as methylation status, to clarify their impact on penetrance and expressivity [47–50]. More broadly, phasing of sequence variants with local methylation status can provide insight into expected allele-specific expression in single-gene disorders.

Beyond methylation, LRS can detect other modifications such as hydroxymethylation and N6-methyladenine, further linking epigenetic regulation to Mendelian disease. As analytic pipelines mature, the integration of genetic and epigenetic information from LRS should refine variant interpretation, improve diagnostic rates, and clarify predictions of prognosis (see Tables 1 and 2) [51, 52].

**Table 2:** More detailed comparisons of characteristics of short read and long read sequencing and potential neurological disorders that could benefit

Application	Challenge with Short-Read Sequencing (SRS)	LRS Advantage (PacBio HiFi/ONT)	Clinical/Neurological disorders with Relevance
Repeat Expansion Disorders	PCR stutter, poor alignment, read length too short; often requires Repeat Primed-PCR or Southern blot. Reliable repeat sizing ~ 50–100 bp	Capable of capturing full STR expansions (even GC-rich loci); resolves size and interruptions. PacBio repeat limit of ~ 15–30 units (trinucleotide) ~ 20–25 kb total span. ONT repeat limit of ~ 6,000–8,000 trinucleotide units, > 2 Mb theoretical span; but limited by raw error rate in repetitive sequence	Huntington disease(OMIM#:143,100),, Fragile X(OMIM#:300,624), myotonic dystrophy(OMIM#160,900), Friedreich ataxia(OMIM#:229,300), SCAs, CANVAS(OMIM#:614,575)
Structural Variants (SVs)	Limited resolution of CNVs, inversions, translocations; misses many large variants. ~ 50 bp minimum; poor for balanced SVs, repetitive regions	Long reads span entire SVs; population studies revealed > 160 k new SVs. PacBio has a ~ 50 bp minimum; excels in complex/segmental duplication regions. ONT has a minimum of ~ 50 bp; best for very large SVs and complex multi-breakpoint events SV classes with greatest LRS benefit—Mobile element insertions, inversions, balanced translocations, complex SVs	Parkinson disease(OMIM#:168,600), Alzheimer disease(OMIM#:168,600), ALS(OMIM#:105,500), autism(OMIM#:209,850), Kabuki syndrome(OMIM#:147,920), DMD(OMIM#:300,377), Pelizaeus-Merzbacher(OMIM#:312,080)
Phasing/Allelic Resolution	Requires trio sequencing or computational inference; error-prone in repetitive regions and highly homologous regions	Direct phasing from single individuals; resolves cis vs. trans variants accurately	Recessive disorders, compound heterozygotes, haplotype-specific risk interpretation
Mitochondrial Genome	Fragmentation loses circularity; PCR bias; poor detection of heteroplasmy and large deletions	Sequencing of full mtDNA molecules; accurate breakpoint detection; improved heteroplasmy analysis	Parkinson disease (OMIM#:168,600), Alzheimer disease (OMIM#:168,600), Huntington disease (OMIM#:143,100), Kearns-Sayre syndrome (OMIM#:530,000), mtDNA deletions
Epigenetic Disorders	Bisulfite sequencing and arrays degrade DNA, introduce bias, and lack allele-specific resolution	Direct detection of methylation and other base modifications at single-molecule level; phasing of variants with methylation	Fragile X syndrome(OMIM#:300,624), Prader–Willi syndrome(OMIM#:176,270), Angelman syndrome(OMIM#:105,830), Friedreich ataxia(OMIM#:229,300), imprinting disorders

## Clinical applications of LRS in case studies

While applications of LRS in the clinical setting are still emerging, published cases already show how LRS can improve ND diagnosis and treatment. In particular, research into ONT LRS for neuromuscular disorders has been productive. One study of 12 suspected cases of DMD (OMIM#:300,377) using LRS were able to find 7 SVs [36]. Other studies have also improved diagnostic yields for DMD and revealed SVs [22, 53–55]. A study for spastic ataxia (OMIM#:108,600) used ONT LRS in 34 patients with a clinical, but not genetic, diagnosis (23 of which had previously had SRS completed). In addition to uncovering the phased results (cis vs. trans) for compound heterozygotes, they were able to genetically diagnose 14 of the patients, 10/14 (71%) of whom had STRs and the other 4/14 (29%) had a SNV detected [56]. These studies demonstrate an improved diagnostic yield using LRS through the detection of SVs, STRs, and SNVs. LRS will be beneficial to deciphering long and complex genes like those in DMD. Many other neuromuscular studies have also found STRs in patients that enabled diagnosis of an autosomal recessive disease diagnoses [50, 57–63]. Others have also used the technique for phasing [62–64].

Beyond neuromuscular disorders, diagnostic improvement in neurodevelopmental disorders has also been achieved with LRS. These have also been instrumental in detecting SVs, STRs, SNVs, and in phasing (see Fig. 2). Some conditions that have been shown to have improved detection using LRS include benign adult familial myoclonic epilepsy (OMIM#:601,068), *CANVAS* (OMIM#:614,575)*,* congenital central hypoventilation syndrome (OMIM#:209,880), Huntington disease (OMIM#:143,100), myotonic dystrophy (OMIM#:160,900), neuronal intranuclear inclusion disease (OMIM#:603,472), spinocerebellar ataxia (OMIM#:164,400), Unverricht–Lundborg disease (OMIM#:254,800), and Fragile X Syndrome [12, 65–67] (Table 3).

**Table 3:** Benefits of long read sequencing in clarifying neurologic disorders

Genetic Mechanism	Challenge with SRS	LRS Contribution	Representative Disorders	References (PMID)
Structural Variants (SVs)	Limited resolution for large insertions/deletions/duplications, inversions, and complex rearrangements	Long reads span breakpoints, detect novel SVs, improve breakpoint accuracy	DMD (OMIM#:300,377), LAMA2-related dystrophy(OMIM#:156,225), Kabuki syndrome (OMIM#:147,920), Pelizaeus–Merzbacher disease(OMIM#:312,080)	35,734,998, 36,198,806, 32,951,359, 37,023,488, 34,018,669, 35,734,998
Repeat Expansions (STRs)	PCR stutter, poor alignment, read length too short to span repeats	Resolves full repeat length and interruptions, even in GC-rich regions	Huntington disease (OMIM#:143,100), Fragile X (OMIM#:300,624), myotonic dystrophy(OMIM#160,900), Friedreich ataxia(OMIM#:229,300), spinocerebellar ataxias (SCAs), CANVAS(OMIM#:614,575), benign adult familial myoclonic epilepsy(OMIM#:601,068)	31,332,380, 33,693,509, 35,148,830, 39,068,203, 37,320,968, 33,807,660, 35,741,732, 35,580,751, 37,158,973, 36,289,212, 31,332,381, 29,507,423, 33,937,879, 40,788,430
Single Nucleotide Variants (SNVs)	May miss pathogenic SNVs in repetitive/complex/dark regions	Detects SNVs alongside phasing, sometimes missed by SRS	Spastic ataxia(OMIM#:108,600), neuronal intranuclear inclusion disease	40,007,153
Phasing/Allelic Resolution	Requires trio sequencing or heavy computational inference; error-prone in repetitive regions	Direct phasing from single individuals; resolves cis vs. trans variants accurately	Spastic ataxia(OMIM#:108,600), recessive neuromuscular disorders, compound heterozygotes	40,007,153, 35,580,751, 37,158,973, 37,932,106
Epigenetic Alterations	Bisulfite sequencing/arrays degrade DNA, lack allele-specific resolution	Direct detection of methylation, allele-specific analysis, phasing with genetic variants	Fragile X (OMIM#:300,624) (CGG repeat expansion with promoter hypermethylation), imprinting disorders (Prader–Willi syndrome (OMIM#:176,270), Angelman syndrome (OMIM#:105,830))	36,289,212, 33,937,879
Mitochondrial Variants/CNVs	Fragmentation and PCR bias obscure deletions/heteroplasmy	Full-length mtDNA reads improve breakpoint resolution and heteroplasmy detection	Kearns–Sayre syndrome (OMIM#:530,000), Parkinson disease (OMIM#:168,600), Alzheimer disease (OMIM#:168,600), Huntington disease (OMIM#:143,100),	37,456,669, 37,642,407

## Challenges and limitations

While LRS is revolutionary in many aspects, like any new technology it also presents challenges. In PacBio HiFi, the double stranded DNA molecule must be circularized (Fig. 1). If the cut site to do so is within a variant, there may be uneven coverage of the region, causing variants to be missed. Additionally, ONT can be particularly computationally complex and expensive. This can result in base calling bottlenecks [44, 68]. Other causes of bioinformatics bottlenecks in both systems include error correction, read alignment, and genome polishing [44, 68]. NDs are, as discussed above, often the result of repeat expansions and structural variants, elongating the variant region and therefore making them particularly vulnerable to these computational limitations.

Beyond technical hurdles, practical limitations constrain clinical rollout. LRS requires specialized infrastructure, including high-throughput sequencers, robust data storage, as well as personnel skilled in molecular genetics and bioinformatics [69]. Building and sustaining commercial-grade sequencing pipelines is expensive and time-consuming. In addition, standardized quality assurance, regulatory frameworks, and comprehensive sequence analysis pipelines are still being developed, making it difficult for smaller institutions to implement LRS at scale. The first step to LRS implementation requires a set standard of use. To develop such standards, we must first evaluate the evidence of its efficacy in diagnostics. From there, questions about scalability and access can be addressed. Costs can vary greatly depending on the equipment, read length, and sample. ONT tends to be less expensive with costs ranging from 3–72$ per Gb and PacBio on the other hand has ranges of 11–86$ per Gb. SRS methods have the cheapest options with a typical range of 2–35$ per Gb [70]. Some institutions have implemented on-site sequencers, which enables a quick turnaround but may not be the most cost-effective approach given the economies of scale achieved by centralized, tertiary labs [71]. Some workflows have been developed to create uniform diagnostic methods using LRS [72, 73]. In addition, quality control methods have been developed to ensure that the technology is being used responsibly. The standard quality control methods used in SRS like FastQC are not yet well suited for LRS. Instead, quality control pipelines like LongQC use the rate of nonsense reads which are sections that can’t be matched to any molecule in the same library. Polishing technologies like DASCRUBBER are also being used [18]. Although LRS has shown promise in identifying pathogenic variants in some NDs, studies remain limited in scale and scope, and broader validation across the full spectrum of NDs is still lacking. Our understanding of LRS’ potential diagnostic power in NDs is growing rapidly but is not yet mature enough for widespread clinical implementation [14, 67]. Further validation studies are needed to establish the sensitivity, specificity, and reproducibility of LRS across diverse variant classes and clinical contexts. Systematic benchmarking of LRS platforms against established diagnostic approaches will help define their clinical utility. This has, in part, caused reimbursement pathways for LRS-based diagnostics to remain limited, further slowing adoption. As a result, access to LRS is largely restricted to major academic centers or pilot projects. This raises concerns about equitable access across healthcare systems [74]. Efforts are underway to address these barriers through the development of smaller sequencers with lower capital costs (e.g., ONT’s MinION/PromethION-2 platforms; PacBio’s Vega platform), integrated analysis pipelines (e.g., ONT’s wf-human-variation; PacBio’s HiFi-human-WGS-WDL), and commercial partnerships that provide sequencing as a service. However, until costs decrease, regulatory frameworks mature, and bioinformatics workflows become more automated, the translation of LRS from research into routine clinical diagnostics will remain gradual [71].

## Ethical considerations

A major problem with LRS is equitable access. While this is also a concern for SRS, the much more limited availability of and rapidly evolving nature of LRS technology creates more profound access inequity. Currently, LRS is mainly available at large academic and medical centers. LRS also still carries a higher cost per kilobase than SRS. Due to its novelty and rapid evolution, LRS is rarely covered by insurance. These factors constrain access to higher-resourced families with the disposable income, travel capability, and/or clinical trial enrollments [75]. Combined, these socioeconomic and geographical limitations risk widening the diagnostic gap as NDs’ clinical management becomes more reliant on LRS. As research and clinical practice expands to include LRS, these limitations must be addressed to ensure the clinical and diagnostic advancements of LRS can benefit all families.

Steps to overcome these disparities include increasing access, reducing costs, improving insurance reimbursement, educating clinicians and counselors, and including diverse populations in both reference datasets and clinical validation studies. Broader deployment of lower-capital LRS platforms (like the handheld MinION and benchtop PromethION-2 and Vega) and LR sequencing-as-a-service laboratories could help to reduce geographic disparities and cost. The type of variants identified is also of note, on one hand, improved resolution may help reclassify variants previously considered uncertain. On the other hand, LRS can identify more complex and previously unobservable genomic features whose clinical significance may not yet be well understood. Variants of unknown significance (VUS) represent a major challenge in both SRS and LRS-based diagnostics. Lessons from SRS have demonstrated that VUS interpretation improves over time through data sharing of genotype–phenotype correlations. VUS require conscientious, informed counseling about expected risk and prognosis. Wider screening with LRS will also increase the discovery of incidental variants unrelated to initial clinical indications. These incidental findings should be carefully disclosed to patients, aiming to minimize the psychosocial impact of an unexpected diagnosis or risk assessment.

## Conclusion and future directions

The existing corpora of research and case reports show that LRS promises diagnostic improvements for NDs. Molecular diagnosis in NDs often requires identifying large SVs and STRs, establishing variant phasing, and/or analyzing mitochondrial genomes. LRS can overcome these longstanding SRS blind spots, enabling diagnoses in previously unresolved cases and improving VUS classification. These advantages are promising: LRS’ combination of long read length with relatively high accuracy should make the technique diagnostically useful once today’s limitations are overcome. At present, LRS’ need for such high-quality samples, restricted availability, computational bottlenecks, and cost limit its deployment as a widespread standard diagnostic. To better understand the impacts of LRS on diagnostics, pilot quality improvement studies could serve to ideate and troubleshoot broader implementation. Future research should focus on longitudinal outcome studies to evaluate how LRS-based diagnoses influence clinical management and diagnosis.
